# Mutant prevention concentration of ozenoxacin for quinolone-susceptible or -resistant *Staphylococcus aureus* and *Staphylococcus epidermidis*

**DOI:** 10.1371/journal.pone.0223326

**Published:** 2019-10-09

**Authors:** Y. López, M. Tato, D. Gargallo-Viola, R. Cantón, J. Vila, I. Zsolt

**Affiliations:** 1 Institute of Global Health of Barcelona, Barcelona, Spain; 2 Department of Clinical Microbiology, Hospital Universitario Ramón y Cajal & Instituto Ramón y Cajal de Investigación Sanitaria (IRYCIS), Madrid, Spain; 3 ABAC Therapeutics, Barcelona, Spain; 4 Department of Clinical Microbiology, Hospital Clinic, School of Medicine, University of Barcelona, Spain; 5 Medical Department, Ferrer Internacional, Barcelona, Spain; University of Mississippi Medical Center, UNITED STATES

## Abstract

Ozenoxacin (OZN) belongs to a new generation of non-fluorinated quinolones for the topical treatment of skin infections which has shown to be effective in the treatment of susceptible and resistant Gram-positive cocci. The mutant prevention concentration (MPC) of ozenoxacin, levofloxacin and ciprofloxacin was determined in quinolone-susceptible and -resistant strains including methicillin-susceptible *S*. *aureus*, methicillin-resistant *S*. *aureus*, methicillin-susceptible *S*. *epidermidis* and methicillin-resistant *S*. *epidermidis* with different profile of mutation in the quinolone resistance determining regions (QRDR). The MPC value of OZN for the methicillin-susceptible *S*. *aureus* strain susceptible to quinolones, without mutations in QRDR, was 0.05 mg/L, being 280-fold lower than that observed with ciprofloxacin and levofloxacin. In methicillin-susceptible and–resistant *S*. *aureus* strains with mutations in the *gyrA* or/and *grlA* genes the MPC of OZN went from 0.1 to 6 mg/L, whereas the MPC of levofloxacin and ciprofloxacin was > 50 mg/L for the same strains. For methicillin-susceptible and–resistant *S*. *epidermidis* the results were similar to those abovementioned for *S*. *aureus*. According to our results, the MPC of OZN was far below the quantity of ozenoxacin achieved in the epidermal layer, suggesting that the *in vivo* selection of mutants, if it occurs, will take place at low frequency. Ozenoxacin is an excellent candidate for the treatment of bacterial infections caused by susceptible and quinolone-resistant staphylococci isolated usually from skin infections.

## Introduction

Staphylococci are among the major groups of bacterial commensals isolated from skin and mucous membranes of humans [1]. In addition, staphylococci is a predominant organism causing infections in both community- and hospital-setting [2]. Specifically, *Staphylococcus aureus* is the most common bacterium associated with skin infections, such as folliculitis and impetigo, affecting children more so than adults worldwide. However, it can also cause skin and soft tissue infections in the hospitals as well as more severe infections such as pneumonia, bacteremia, endocarditis and osteomyelitis [3, 4]. On the other hand, *Staphylococcus epidermidis* is the most common microorganism on normal skin microbiota, being considered today as an important opportunistic pathogen, and the most common source of infections on indwelling medical devices [5, 6].

The treatment for these infections includes topical and/or oral antimicrobial agents, according to the severity of the infection and damaged skin surface [7]. Topically administered antibacterial agents include mupirocin, fusidic acid and retapamulin. Unfortunately, an increasing number of Gram-positive pathogens, especially methicillin-resistant *S*. *aureus* (MRSA), have developed resistance to topical antimicrobial agents typically used in clinical practice, potentially limiting its overall efficacy [8]. Recently, a community-acquired methicillin-resistant *S*. *aureus* strain carrying a plasmid conferring resistance to mupirocin and chlorhexidine has been reported, which reinforce the need for potential alternatives to threat infections caused by these type of strains [9].

Nowadays, ozenoxacin (OZN) is a more recent alternative for topical treatment of infection skin, with excellent clinical benefit in two recent Phase III trials [10]. OZN belongs to a new generation of non-fluorinated quinolones, demonstrating excellent antibacterial activity *in vitro* against Gram-positive cocci including resistant strains to other quinolones and low capacity to select resistant mutant strains [11–15].

Quinolones bind to the complex DNA-DNA gyrase and DNA-topoisomerase IV, both of these enzymes are involved in bacterial DNA synthesis [16]. However, the bactericidal effect of these antibiotics is related, at least in part, to the accumulation of reactive oxygen species (ROS) and oxidative damage of several macromolecules [17–19]. The main mechanism of resistance to quinolones in staphylococci is associated with mutations in a specific region of the *gyr*A (encoding the A subunit of DNA gyrase) and *grl*A (encoding the A subunit of topoisomerase IV) genes called the Quinolone Resistance-Determining Regions (QRDR). Several mutations in a step-wise resistance acquisition can provide high-level of quinolone resistance [20].

The appearance of resistant mutants in an infectious process is high and usually occurs randomly and spontaneously [21]. For this reason, a drug concentration threshold above which the bacterial cells require the presence of two or more mutations for their survival has been designated. The antibiotic concentration above this threshold has been defined as the mutant prevention concentration (MPC), which corresponds to the MIC of the less susceptible subpopulation and should severely restrict the selection of resistant mutants [22]. In the present study, we have determined the mutant prevention concentration of ozenoxacin compared with other quinolones, such as levofloxacin and ciprofloxacin for *S*. *aureus* and *S*. *epidermidis* clinical isolates associated with skin infection.

## Materials and methods

Fifteen quinolone-resistant and -susceptible strains with different genetic profiles in QRDR region (previously characterized by PCR and sequencing) were analyzed. The strains were selected from a previous study and obtained from the Clinical Microbiology Laboratory at the Hospital Clinic in Barcelona, Spain [12]. The strains included: methicillin-susceptible *S*. *aureus* (MSSA) (5 strains), methicillin-resistant *S*. *aureus* (3 strains), methicillin-susceptible *S*. *epidermidis* (MSSE) (3 strains) and methicillin-resistant *S*. *epidermidis* (4 strains).The MPC of OZN (Ferrer Laboratories), levofloxacin (LVX) and ciprofloxacin (CIP) (Sigma-Aldrich, St. Louis, MO) were performed in triplicate and we used the technique described by our colleagues [23] with some modifications. Briefly, the microorganisms were cultured in Muller Hinton broth (MHB, Becton Dickinson, Sparks, MD) and incubated for 24 h. Then, 1/10 dilution was made in fresh culture medium to be subsequently incubated for 4 h at 37°C with shaking. Aliquots of 1 ml were concentrated by centrifugation at 5,000 x g for 5 min, resuspended in 100 μl fresh culture medium (approximately 10^10^−10^11^ cfu/mL) and inoculated onto Mueller-Hinton agar (MHA, Oxoid, UK) plates containing increasing concentrations of fluoroquinolones from concentrations lower than the MIC of each microorganism. The inoculum size was confirmed by serial dilutions and plating on drug-free medium. The inoculated plates were incubated for 24–48 h at 37°C and screened visually for growth. The MPC value corresponds to the concentration that does not allow the recovery of bacterial colonies.

## Results and discussion

According to the results obtained in this study, OZN has demonstrated lower values of MPC compared to LVX and CIP in all strains of *S*. *aureus* and *S*. *epidermidis* included in the study, as shown in Tables 1 and 2.

**Table 1:** Activities of ozenoxacin, levofloxacin and ciprofloxacin against isolates of *S*. *aureus*.

Isolates	Mutation QRDR		Antimicrobial Agent	MIC mg/L	MPC ^1^ mg/L
	*gyrA*	*grlA*			
**MSSA**					
4–149	WM ^2^	WM	OZN	0.0039	0.05
			LEV	0.25	14
			CIP	0.38	14
440	S84L	WM	OZN	0.125	0.8
			LEV	64	110
			CIP	64	125
8901	WM	S87L	OZN	0.008	0.1
			LEV	0.5	14
			CIP	2	20
176	S84L/S85P	WM	OZN	0.5	1.2
			LEV	128	400
			CIP	256	550
51	S84L/S85P	S80Y/E84G	OZN	2	6
			LEV	64	450
			CIP	256	650
**MRSA**^**3**^					
108	S84L	WM	OZN	0.06	0.6
			LEV	16	85
			CIP	16	75
823	S84L	WM	OZN	0.125	0.6
			LEV	64	200
			CIP	256	400
126	S84L/E88K	S80F/E84V	OZN	2	6
			LEV	512	700
			CIP	256	350

^1^ Mutant prevention concentration (MPC). This parameter was defined to characterize the capacity to prevent/severely restrict the emergence of drug-resistant mutants [22].

^2^ WM, without mutation in the QRDR

^3^ No data on MRSA WM are available

**Table 2:** Activities of ozenoxacin, levofloxacin and ciprofloxacin against isolates of *S*. *epidermidis*.

Isolates	Mutation QRDR		Antimicrobial Agent	MIC mg/L	MPC^1^ mg/L
	*gyrA parC*				
**MSSE**					
HCL43141	WM ^2^	WM	OZN	0.0078	0.025
			LEV	0.125	0.7
			CIP	0.19	1.5
HCL 46313	WM	S80Y	OZN	0.0078	0.025
			LEV	0.125	0.6
			CIP	0.19	0.9
56	S84F/E88K	S80F	OZN	2	6
			LEV	512	750
			CIP	128	300
**MRSE**					
7602	WM	WM	OZN	0.03	0.05
			LEV	0.5	2
			CIP	1	4
6902	S84F	WM	OZN	0.031	0.1
			LEV	2	4
			CIP	2	20
FG012	S84F	S80F	OZN	0.12	0.3
			LEV	32	80
			CIP	128	250
FG013	S84Y/E88K	S80F/D84Y	OZN	2	6
			LEV	512	900
			CIP	128	250

^1^ Mutant prevention concentration (MPC). This parameter was defined to characterize the capacity to prevent/severely restrict the emergence of drug-resistant mutants [22]

^2^ WM, without mutation in the QRDR

The MPC value of OZN for the MSSA strain susceptible to quinolones, without mutations in QRDR, was 0.05 mg/L, being 280-fold lower than that observed with CIP and LVX (14 mg/L). On the other hand, MSSA strain with 4 mutations in the QRDR showed a MPC value of OZN of 6 mg/L, being more than 70-fold lower in comparison with other studied quinolones. Similar results were observed in the group of MRSA strains, although unfortunately there are no data available in MRSA susceptible to quinolones, without mutations in the QRDR. MRSA with 4 mutations in the QRDR, showed a maximum value of MPC of OZN of 6 mg/L, which was considerably lower than the MPC values of LVX and CIP (700 and 350 mg/L, respectively).

Results of the MPC obtained for strains of *S*. *epidermidis* showed a similar behavior to that observed in the strains of *S*. *aureus*. The MPC value of OZN was 0.025 and 0.05 mg/L, respectively, for the MSSE and MRSE strains susceptible to quinolones, without QRDR mutations, being lower than that observed with LVX (0.7–2 mg/L) and CIP (1.5–4 mg/L). On the other hand, the MPC of OZN for MSSE and MRSE strains resistant to quinolones with 3 and 4 mutations in the QRDR was 6 mg/L in both groups of strains, being significantly lower than LVX and CIP, whose value of concentration for preventing the appearance of resistant mutants was 750 and 900 mg/L and 300 and 250 mg/L, respectively.

According to our observations, after exposing a high bacterial inoculum (10^11^cfu/mL) to increasing concentrations of OZN, LVX or CIP, it was possible to recover subpopulations that survived at a higher concentration than the initial MIC in both groups of methicillin- and quinolone-susceptible and -resistant staphylococci strains. However, if we compare the MPC values of both species, we observed that OZN value fluctuated between 0.025 and 6 mg/L, being the last value observed in a strain with 4 mutations in the QRDR. In other words, only 6 mg/L of OZN are needed to inhibit the growth of the most resistant subpopulation in a high bacterial inoculum.

On the other hand, if we compare the MPC value of the comparative quinolones in the same group of strains, we observe that this value fluctuated between 0.6 and 900 mg/L for LVX and 0.9 and 650 mg/L for CIP, which are considerably higher values than those observed with OZN.

Several studies in quinolones of fourth generation have reported generally similar results to ours although with some discrepancies for some strains. For example, Metzler and colleagues [24] reported the MPCs values of different fluoroquinolones for MSSA and MRSA strains. In this study, LVX MIC_90_ for MSSA strains was 0.25 mg/L and the MPC_90_ of 1 mg/L, being lower than the MPC of LVX found in our study for wild-type MSSA strain (without mutation in QRDR). However, the MIC_90_ of LVX for MRSA was >16 mg/L and the MPC_90_ of 128 mg/L, being higher than the one strain found in our study (MIC of LVX of 16 mg/L and MPC of 85 mg/L). Additionally, studies described by Liu and colleagues [25] showed that when the MIC of LVX for MRSE strains was 0.25 mg/L, the obtained MPC value was between 4–8 mg/L, similar to the results in our study. On the other hand, studies with delafloxacin a novel fluoroquinolone, showed that the MPC values ranged from one to four times the initial MIC and were markedly lower (8- to 32-fold) than the MPCs for the other quinolones included in that study. This fact is an excellent characteristic for an antimicrobial agent. However, as the analysis was only performed on MRSA strains, it is not possible to completely compare with our results [26].

In conclusion, OZN shows a strong ability to restrict the development of resistant strains as following only a slight increase in OZN concentration, the eradication of the most resistant subpopulations with possible multiple mutations in the QRDR occur. This suggests that the *in vivo* mutant selection, if it occurs, will take place at low frequency. In addition, the MPC values found in our study was in all the cases below the concentration of ozenoxacin achieved in the epidermis (of 22 mg/L after 3 days, twice a day application [27], a quantity of OZN far higher than the maximal range of MIC and MPC for resistant staphylococci strains detected in all the performed *in vitro* studies. These results are probably due to its potent activity linked to the strong inhibition of both protein targets and to the rapid accumulation inside bacteria [11,28, 29]. For this reason, OZN is an excellent candidate for the treatment of bacterial infections caused by susceptible and methicillin-resistant and/or quinolone-resistant Gram-positive pathogens isolated from skin infections.

## Supporting information

S1 Table (XLSX)

## References

[1] Marsilio F, Di Francesco CE, Di Martino B. Coagulase-Positive and Coagulase-Negative Staphylococci Animal Diseases. *Pet-To-Man Travel Staphylococci* 2018; 43–50.

[2] Gad Gamal Fadl M., El-Ghafar Abd El-Ghafar F. Abd, El-Domany Ramadan A. A., Hashem Zeinab Shawky. Epidemiology and antimicrobial resistance of staphylococci isolated from different infectious diseases. *Braz. J. Microbiol*. 2010 6 28; 41 (2): 333–344 10.1590/S1517-838220100002000012 24031501 PMC3768675

[3] Maina EK, Kiiyukia C, Wamae CN, Waiyaki PG and Kariuki S. Characterization of methicillin-resistant *Staphylococcus aureus* from skin and soft tissue infections in patients in Nairobi, Kenya. *Int J Infect Dis* 2013; 17: e115–9 10.1016/j.ijid.2012.09.006 23092752

[4] Sladden MJ, Johnston GA. Common skin infections in children. *Aust Fam Physician* 2004; 329: 95–99. 10.1136/bmj.329.7457.95 PMC449820 15242915

[5] Otto M. Staphylococcus epidermidis—the ‘accidental’ pathogen. *Nat Publ Gr* 2009; 7: 555–567. 10.1038/nrmicro2182 PMC2807625 19609257

[6] Pereira LB. Impetigo—Review. *An Bras Dermatol*. 2014 Mar-Apr; 89(2): 293–299. 10.1590/abd1806-4841.20142283 24770507 PMC4008061

[7] Koning S, van der Sande R, Verhagen AP, van Suijlekom-Smit LW, Morris AD, Butler CC., et al Interventions for impetigo. *Cochrane Database of Systematic Reviews*; 2012 1 18;1:CD003261. 10.1002/14651858.CD003261.pub3 PMC7025440 22258953

[8] Vila J, Hebert AA, López Y, García-Castillo M, Cantón R, Torrelo A, et al Ozenoxacin: a review of preclinical and clinical efficacy. *Expert Rev Anti Infect Ther* 2019; 17: 159–168. 10.1080/14787210.2019.1573671 30686133

[9] Copin R, Sause WE, Fulmer Y, Balasubramanian D, Dyzenhaus S, Ahmed JMet al. Sequential evolution of virulence and resistance during clonal spread of community-acquired methicillin-resistant *Staphylococcus aureus*. *Proc Natl Acad Sci*. 2019; 116(5): 1745–1754. 10.1073/pnas.1814265116 30635416 PMC6358666

[10] Hebert AA, Albareda N, Rosen T, Torrelo A, Grimalt R, Rosenberg N, Zsolt I MX. Topical Antibacterial Agent for Treatment of Adult and Pediatric Patients With Impetigo: Pooled Analysis of Phase 3 Clinical Trials. *Drugs Dermatol* 2018; 17: 1046–1052. 30365584

[11] Yamakawa T, Mitsuyama J, Hayashi K. *In vitro* and *in vivo* antibacterial activity of T-3912, a novel non-fluorinated topical quinolone. *J Antimicrob Chemother* 2002; 49: 455–465. 10.1093/jac/49.3.455 11864945

[12] López Y, Tato M, Espinal P, Garcia-Alonso F, Gargallo-Viola D, Cantón R, et al *In vitro* activity of Ozenoxacin against Gram-positive bacteria susceptible and resistant to other quinolones. *Antimicrob Agents Chemother* 2013 12; 57 (12): 6389–92. 10.1128/AAC.01509-13 24080666 PMC3837899

[13] López Y, Tato M, Espinal P, Garcia-Alonso F, Gargallo-Viola D, Canton R, et al *In vitro* selection of mutants resistant to ozenoxacin compared with levofloxacin and ciprofloxacin in Gram-positive cocci. *J*. *Antimicrob*. *Chemother* 2014 1; 70 (1):57–61. 10.1093/jac/dku375 25261416

[14] Canton R, Morrissey I, Vila J, Tato M, García-Castillo M, López Y, et al Comparative i*n vitro* antibacterial activity of ozenoxacin against Gram-positive clinical isolates. *Future Microbiol* 2018; 13: 3–19. 10.2217/fmb-2017-0289 29745242

[15] Kanayama S, Ikeda F, Okamoto K, Nakajima A, Matsumoto T, Ishii R, et al In vitro antimicrobial activity of ozenoxacin against methicillin-susceptible Staphylococcus aureus, methicillin-resistant S. aureus and Streptococcus pyogenes isolated from clinical cutaneous specimens in Japan. *J Infect Chemother* 2016; 22: 720–723. 10.1016/j.jiac.2016.03.006 27091753

[16] Fàbrega A, Madurga S, Giralt E, et al Mechanism of action of and resistance to quinolones. *Microb Biotechnol* 2009; 2: 40–61. 10.1111/j.1751-7915.2008.00063.x 21261881 PMC3815421

[17] Dwyer DJ, Kohanski MA, Hayete B, Collins JJ. Gyrase inhibitors induce an oxidative damage cellular death pathway in Escherichia coli. *Mol Syst Biol*. 2007; 3:91 10.1038/msb4100135 17353933 PMC1847949

[18] Zhao X, Hong Y, Drlica K. Moving forward with reactive oxygen species involvement in antimicrobial lethality. *J Antimicrob Chemother* 2015; 70: 639–642. 10.1093/jac/dku463 25422287 PMC4319487

[19] Hong Y, Zeng J, Wang X, Drlica K and Zhao X. Post-stress bacterial cell death mediated by reactive oxygen species. *Proc Natl Acad Sci* 2019; 116 (20): 10064–10071. 10.1073/pnas.1901730116 30948634 PMC6525477

[20] Hooper DC, Jacoby GA. Mechanisms of drug resistance: quinolone resistance. *Ann N Y Acad Sci* 2015; 1354: 12–31. 10.1111/nyas.12830 26190223 PMC4626314

[21] Drlica K. The mutant selection window and antimicrobial resistance. *J Antimicrob Chemother* 2003; 52: 11–17. 10.1093/jac/dkg269 12805267

[22] Drlica K, Zhao X. Mutant Selection Window Hypothesis Updated. *Clin Infect Dis* 2007; 44: 681–688. 10.1086/511642 17278059

[23] Gebru E, Choi MJ, Lee SJ, Damte D and Park SC. Mutant-prevention concentration and mechanism of resistance in clinical isolates and Enrofloxacin/ marbofloxacin-selected mutants of *Escherichia coli* of canine origin. *J Med Microbiol* 2011; 60: 1512–1522. 10.1099/jmm.0.028654-0 21596912

[24] Metzler K, Hansen GM, Hedlin P, Harding E, Drlica K, and Blondeau JM. Comparison of minimal inhibitory and mutant prevention drug concentrations of 4 fluoroquinolones against clinical isolates of methicillin-susceptible and -resistant Staphylococcus aureus. *Int J Antimicrob Agents* 2004; 24: 161–167. 10.1016/j.ijantimicag.2004.02.021 15288315

[25] Liu L, Zhu Y, Hu L, Cheng J, Ye Y, and Li J. Comparative study of the mutant prevention concentrations of vancomycin alone and in combination with levofloxacin, rifampicin and fosfomycin against methicillin-resistant *Staphylococcus epidermidis*. *J* Antibiot (Tokyo) 2013; 66: 709–12. 23981959 10.1038/ja.2013.87

[26] Remy JM, Tow-Keogh CA, McConnell TS, Dalton JM, and Devito JA. Activity of delafloxacin against methicillin-resistant *Staphylococcus aureus*: resistance selection and characterization. *J Antimicrob Chemother* 2012; 67: 2814–20. 10.1093/jac/dks307 22875850

[27] Gropper S, Albareda N, Santos B, Febbraro S. Skin tissue exposure of once- versus twice-daily topical ozenoxacin 2% cream: a Phase I study in healthy volunteers. Future Microbiol. 2014;9(8 Suppl):S17–22. 10.2217/fmb.14.83 25209520

[28] Y. López, Tato M, Cantón R and Vila J. The effect of Ozenoxacin and other quinolones on topoisomerases type II from *Staphylococcus aureus* and *Escherichia coli* Present *53 rd Intersci Conf Antimicrob Agents Chemother (ICAAC)*, 2013 sep 10–13, Denver, USA Abstr C1-522c.

[29] López Y, García-Castillo M, Garcia-Fernandez S, Gargallo-Viola D, Zsolt I, Cantón et al Acumulación de Ozenoxacino y otras quinolonas en bacterias Gram Positivas. *[Abstract Spanish]* Abst 0280 *Enferm Infecc Microbiol Clin* 2018;36 (Espec Cong 1)146.

